# Ave Maria and Visions of Children: Atypical Charles Bonnet Syndrome or Two Coexisting Deafferentation Phenomena?

**DOI:** 10.7759/cureus.3191

**Published:** 2018-08-23

**Authors:** Adriana Y Koek, Patricio S Espinosa

**Affiliations:** 1 Clinical Biomedical Science, Florida Atlantic University Charles E. Schmidt College of Medicine, Boca Raton, USA; 2 Neurology, Marcus Neuroscience Institute, Boca Raton Regional Hospital, Boca Raton, USA

**Keywords:** charles bonnet syndrome, visual hallucinations, musical hallucinations, macular degeneration, visual impairment, atypical charles bonnet syndrome

## Abstract

Charles Bonnet syndrome (CBS) refers to the experience of visual hallucinations in the context of visual impairment. The underlying pathology may be localized anywhere along the visual pathway from the eye itself to visual cortical centers. It is sometimes compared to phantom limb syndrome; both involve decreased sensory input, as in loss of a limb or declining vision, resulting in overactivity in areas of the brain controlling sensory perception. Definitive diagnostic criteria are still lacking and may vary by discipline. However, the following features are generally agreed upon: visual hallucinations, impaired vision, and intact cognition and insight. Psychiatric symptoms, cognitive decline, and hallucinations of other sensory modalities are often excluded, although this remains an area of debate. Certain non-classic cases of CBS have inspired the designation of atypical CBS, which encompasses a wide spectrum of sensory experiences and associated symptoms. Auditory hallucinations in the hearing-impaired, a well-described phenomenon thought to have a similar pathogenesis, share with CBS the important risk factor of increased age. In patients experiencing both types of hallucinations with deterioration in both sensory domains, the distinction between a CBS variant and two independent processes may not be straightforward. In addition to the ongoing diagnostic dilemma posed by multimodal hallucinations, these phenomena tend to be underreported by patients likely due to concern that they will be diagnosed with mental illness. Although many patients with this condition are indifferent to it, some suffer distress from their hallucinations and would benefit from recognition, reassurance, and in some cases correction of the underlying cause or pharmacologic treatment. Here we present the case of an elderly woman with a history of macular degeneration and chronic hearing loss who experienced complex auditory and visual hallucinations surrounding an episode of severe anxiety. We postulate that her anxiety acted as a precipitant to her hallucinatory experiences and may partially explain their abrupt onset in the absence of other clear pathologic processes. This case serves to reinforce CBS as a possible etiology of visual hallucinations in the elderly population, while also generating discussion of how to classify her particular set of symptoms.

## Introduction

Charles Bonnet was an eighteenth-century Swiss philosopher and naturalist who first described the occurrence of complex hallucinations in the visually impaired after his blind grandfather reported seeing visions of people, birds, buildings, and patterns. Hallucinations can arise following a lesion at any point along the visual pathway and from a variety of pathological processes, with age-related macular degeneration (AMD) representing the most commonly associated pathology [1]. CBS is a far more common phenomenon than many physicians and patients realize. Lack of awareness of its nature and prevalence may prevent recognition and treatment of an otherwise benign condition. Below we describe the case of a patient with distressing visual hallucinations likely indicating CBS, which serves as a reminder to consider this in the differential diagnosis of hallucinations, especially in those who are elderly or who suffer from visual or auditory loss.

## Case presentation

This is the case of a 101-year-old Caucasian female with a past medical history of hypertension, melanoma, chronic hearing loss, and macular degeneration who was hospitalized for hypertensive urgency. Her blood pressure on arrival was 205/94, without physical exam or laboratory evidence of end organ damage. On the evening of her second hospital day, she reported frightening auditory and visual hallucinations, and neurology was consulted to evaluate her. She described hearing the sound of drums beating and seeing worms in a container of candy. On further questioning, she admitted to experiencing both visual and auditory hallucinations for several weeks preceding her hospitalization, with the latter arising first. Her visual hallucinations began, and her auditory hallucinations worsened, following an episode of intense anxiety that she described as “body shakes” lasting ten minutes and resolving spontaneously. She had never experienced anything similar in the past, and she denied any associated tongue biting, urinary or fecal incontinence, and confusion. She attributed her anxiety to a pain in her right eye, which she was concerned could be cancerous. She had been recently treated for blepharitis and had no abnormalities on follow-up with her ophthalmologist. The “visions” she described lasted seconds to minutes and usually occurred in the right peripheral field of vision, although she was unsure whether they were present in one or both eyes. They initially consisted of “grey blobs” and “creatures” that stood beside her while she was watching television in the evenings and later became more human-like. Looking directly at these figures or turning on the light made them disappear. While in the hospital, she saw a young girl in a ballerina costume standing beside her and several men dressed in uniform “on the walls and hanging from the ceiling”. She also described “snow” or “dust” coming from the walls. When asked about hallucinations in the past, she said that several years ago she would occasionally see faces just before falling asleep, especially in the setting of emotional “excitement”. She believed this might have been related to the fact that she was a serious artist and frequently painted human faces. She denied hallucinations upon waking, daytime sleepiness, and falling asleep unexpectedly. She denied recent changes in sleep and said she had always slept well at night. Her auditory hallucinations came in the form of music, usually well-recognized melodies such as "America the Beautiful" and "Ave Maria” sung by a man's voice or played on a piano. She heard them in both ears and they were present constantly throughout the day. She occasionally heard voices speaking that were unintelligible except for one time when she heard “she’s a teacher, she teaches English”. She and her family both noticed that the hallucinations worsen in the setting of anxiety or other emotional disturbance. She had no known history of psychiatric illness and had never seen a psychiatrist. On review of systems, she admitted to right eye pain, gradual hearing and vision loss, anxiety, and sadness. She denied headache, dizziness, vertigo, weakness, numbness, tingling, diplopia, photophobia, tinnitus, fever, fatigue, weight change, and memory change.

General examination revealed a mildly anxious elderly woman. On neurological exam, the patient was alert and fully oriented. She scored 29/30 on a Mini Mental State Examination, and 25/30 on the Montreal Cognitive Assessment, with 0/5 points given for delayed recall, although she recalled three with category cue and the remaining two with multiple choice cue. A PHQ-9 gave a score of 2/27. Examination of the cranial nerves revealed full visual fields, decreased visual acuity, and diminished hearing bilaterally with right worse than left on finger rub test. Cranial nerves were otherwise intact. She walked with a walker, and her gait unsupported was unsteady and cautious. The remainder of the neurological exam was unremarkable.

**Figure 1:**
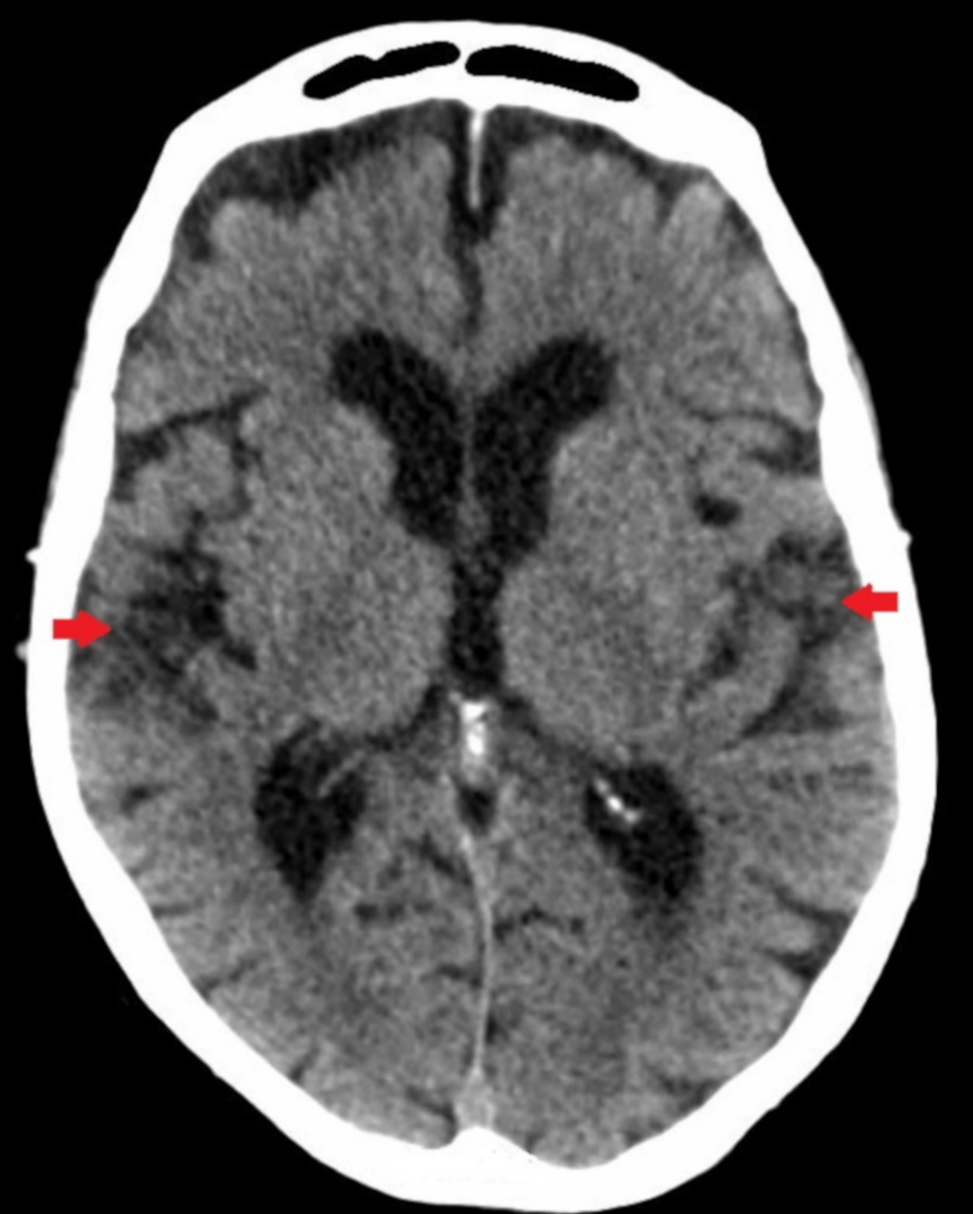
Computed tomography scan of the brain demonstrating mild parietotemporal atrophy.

Computed tomography (CT) scan of the brain without contrast was notable for atrophy in the parietotemporal areas bilaterally, with no evidence of acute infarct, hemorrhage, or mass effect (Figure 1).

She was started on quetiapine at 12.5 mg every evening and discharged once her blood pressure was controlled. At her first follow-up appointment two weeks later, she reported partial improvement in her symptoms. She also noted additional types of hallucinations, including a checkered pattern on the walls and floor and a human arm holding a salt shaker. The arm appeared in her periphery while she was eating breakfast but disappeared when she looked directly at it. She also described intermittent headaches, which she believed to be a manifestation of anxiety, that begin in various locations and become diffuse. Her family reported that she tended to be anxious at baseline with increased anxiety surrounding these sensory experiences. She and her family reported a noticeable decrease in her sense of disturbance upon receiving reassurance that her hallucinations are benign and do not represent mental illness, as well as learning methods to minimize them.

## Discussion

This patient’s complex visual hallucinations, history of AMD, level of insight, and preserved cognition are suggestive of CBS. CBS can occur following any visual insult, acute or chronic, although is most commonly associated with intrinsic ocular disease such as AMD, cataracts, glaucoma, and diabetic retinopathy. Other etiologies to be considered in evaluating a patient with suspected CBS include stroke, seizure, degenerative disease, and malignancy. It is more commonly observed in the elderly population given the age-dependence of many types of chronic visual loss. The prevalence of CBS in patients with visual loss is generally reported to be between 10% and 40%, with the highest prevalence occurring in AMD [2-4]. Not all patients with age-related visual loss experience hallucinations, and in those who do there is no specific threshold of vision impairment at which they arise, although the likelihood increases with the severity of visual impairment [1]. These phenomena are underreported and underdiagnosed, likely owing to the fear of receiving a psychiatric diagnosis and incurring its associated stigma.

There are several standing hypotheses regarding the pathogenesis of hallucinations in CBS, two of the most prominent being the deafferentation and release hypotheses [1,5-7]. The deafferentation hypothesis suggests that loss of visual input alters synaptic function downstream of the affected area, leading to increased excitability and manifesting as visual experiences in the absence of an external stimulus. The release hypothesis, while similarly requiring a loss of afferent visual signals, suggests that their absence releases visual cortical areas from the normal regulatory effect of external visual stimuli. Neuroimaging studies have demonstrated increased activity in the primary visual cortex, secondary visual cortex, and visual association cortices during visual hallucinations in patients with CBS [8-11].

Many of this patient’s symptoms illustrate classic CBS, while others are less typical and prompt consideration of how to define her unique combination of symptoms. The hallucinations of CBS may be simple or complex and can be stereotyped, although there is uncertainty regarding the validity and usefulness of this feature. This woman’s hallucinations of human-like figures, a human body part, children, and geometric patterns on the wall and floor are among those most commonly reported in CBS [5]. The fact that her hallucinations worsen while sitting in a dark room at night and improve in bright light is consistent with the hypotheses of increased excitability or visual release with lack of visual input, as well as reports of sensory deprivation as a risk factor [1,5]. The relationship between her hallucinations and emotional disturbance may suggest stress as an exacerbating factor, which has been observed in some cases [5,12]. The faces she described seeing years earlier just before falling asleep could represent an early presentation of her current condition given their association with emotional “excitement”. This may also help to explain the relatively abrupt onset of her visual hallucinations and worsening of her auditory hallucinations following an episode of severe anxiety. Acute presentations are possible in the setting of acute vision loss, as in the case of infection or cerebral infarct, although this was unlikely in her case given her negative CT scan and lack of clinical signs of infection or other acute process. Further damage to her vision during her hypertensive crisis is possible and might explain the change in nature of her hallucinations while in the hospital, although she reported no changes in visual acuity and no new abnormalities were noted on her fundoscopic exam. Her CT scan showed no appreciable pathology along the central visual pathway or occipital lobes, making the most likely explanation for her hallucinations the visual loss from her AMD.

This case is distinguished by the coexistence of auditory and visual hallucinations, for which there are several potential explanations. By certain definitions, multimodal hallucinations preclude a diagnosis of CBS. However, the designation “atypical” has been introduced in cases of suspected CBS with non-classic features such as poor insight, cognitive decline, history of psychiatric illness, and presence of other hallucinatory modalities [13-16]. Atypical CBS may also include unusually high or persistent stress in response to the hallucinations, which may have been manifested in this patient as ongoing anxiety toward her hallucinatory experiences prior to receiving adequate reassurance. Aside from these two features, her presentation is consistent with a typical picture of CBS. She understood that her hallucinations did not reflect reality, and her normal cognitive testing ruled out neurocognitive decline. Further, the absence of psychiatric history or other significant psychotic or mood symptoms made a primary psychiatric diagnosis unlikely.

While her presentation shares some features with atypical CBS, this distinction merely describes observed clusters of signs and symptoms without necessarily addressing the precise etiology of her auditory hallucinations. Her history of chronic hearing loss and continuous perception of familiar songs are consistent with musical hallucinosis, a phenomenon analogous to the visual hallucinations of CBS. These types of auditory hallucinations are thought to arise by a similar mechanism of increased excitation in the auditory cortex on a background of hearing impairment and may follow a lesion anywhere along the auditory pathway, from the inner ear to the brain [17,18]. They are related in pathogenesis to tinnitus, but their complex and semantic nature may indicate involvement of additional cortical areas, such as those involved in language and memory [19]. Her hearing loss is likely attributable to presbycusis, although some degree of cortical hearing loss cannot be ruled out given evidence on her CT scan of parietotemporal atrophy encompassing the superior temporal gyrus, the location of the primary auditory cortex. This and other cortical areas have been implicated in the functional neurological basis of musical hallucinations based on functional magnetic resonance imaging (fMRI) and other neuroimaging studies [18-20]. We suspect her clinical picture illustrates a case of classic CBS in parallel with musical hallucinosis; while sharing a similar underlying mechanism, they likely arose independently of each other from pathology in different sensory pathways.

This patient improved minimally with quetiapine in terms of severity and frequency of her hallucinations. However, she reported substantial improvement in the anxiety she felt surrounding these experiences once her diagnosis was explained, and she was reassured of its benign nature. Pharmacological treatment options for CBS are limited and primarily derived from anecdotal evidence. Inconsistent results have been reported with antipsychotics, antidepressants, and anticonvulsants [4-6]. Aside from medications, a few interventions may help, including educating patients about the condition and recommending avoidance of sensory and social deprivation. Patients with CBS may experience relief by looking directly at hallucinated objects, blinking rapidly when hallucinations appear, and using bright light.

## Conclusions

Visual and auditory hallucinations in patients with sensory impairment are more prevalent than many realize, affecting a significant proportion of people with age-related sensory impairment. Patients may be reluctant to report symptoms lest they be labeled as mentally ill, and healthcare providers may fail to ask about or follow up on such symptoms due to lack of awareness. This patient with visual and auditory hallucinations, each likely arising from chronic sensory impairment, serves as a reminder to keep non-psychiatric etiologies in mind when evaluating patients with hallucinations in the setting of diminished vision or hearing, especially in the elderly. Increased understanding of the pathogenesis and prevalence of these phenomena may facilitate a nonjudgmental approach to any patient with hallucinations and the opportunity to provide reassurance and appropriate treatment. Patients should be asked about such experiences and encouraged to share them, as this may expedite recognition of benign causes of hallucinations and thwart needless emotional discomfort. Given the interplay between neurological and psychiatric components, patients with presumed musical hallucinosis or CBS should receive adequate workup in each field and be followed closely for ongoing or new distress.
